# Sustainable ruminant livestock transformation through the Global Farm Platform

**DOI:** 10.1093/af/vfag019

**Published:** 2026-08-11

**Authors:** M Jordana Rivero, Joshua Onyango, Tommy Boland, Claire Morgan-Davies, Michael R F Lee

**Affiliations:** Rothamsted Research, North Wyke, Devon, UK; Harper and Keele Veterinary School, Newport, Shropshire, UK; School of Agriculture and Food Science, University College Dublin, Belfield, Dublin, Ireland; SRUC, School of Natural and Social Sciences, Hill & Mountain Research Centre, Crianlarich, UK; Harper Adams University, Newport, Shropshire, UK

**Keywords:** Hub-and-Spoke, Sustainable livestock systems, Sustainable livestock transformation, Sustainable Development Goals, Livestock resilience

Implications

- The Global Farm Platform’s hub-and-spoke network effectively bridges research hubs with commercial and smallholder spokes to accelerate sustainable ruminant livestock transformation worldwide.
- Integration of genomics, long-term research, digital tools, and regional innovations enables enhanced productivity, climate resilience, and environmental benefits while supporting farmer livelihoods.
- This Special Issue demonstrates that collaborative GFP approaches provide a scalable, practical roadmap for sustainable livestock systems.

Ruminant livestock systems are an integral part of agricultural and rural communities globally which provide high quality nutritious food and a plethora of other socio-economic services. However, the impact that ruminant livestock systems can have on climate volatility, land use and biodiversity loss, soil degradation, and water scarcity and quality, calls into focus their future sustainability, especially considering rising global demand for nutritious food. As such there is an urgent need to globally reduce the environmental footprint of ruminant livestock systems while supporting rural livelihoods and maintaining/enhance animal protein production. In response, the Global Farm Platform (GFP; globalfarmplatform.org, Figure 1), launched in 2014 and recently recognized by the FAO for excellence in sustainable livestock transformation, offers a proven, scalable solution. At its core is a hub-and-spoke network that connects research lighthouse farms (hubs) as living laboratories connected to commercial farms and smallholders (spokes). This bidirectional model translates cutting-edge science into context-specific applied innovations, empowering farmers as co-innovators, and delivers measurable gains in productivity, resilience, and public goods such as carbon sequestration, biodiversity recovery, and landscape stewardship.

**Figure 1.**
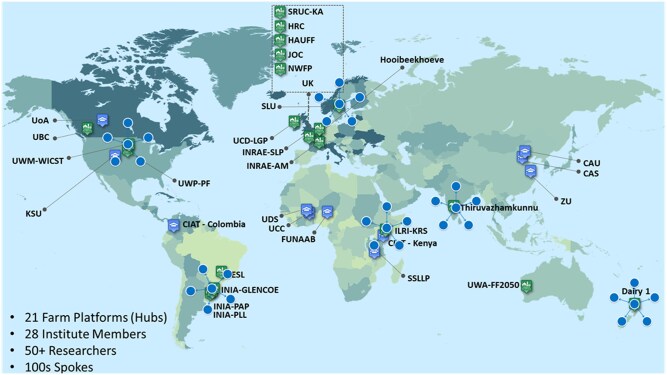
The Global Farm Platform’s hub-and-spoke network: Connecting research hubs to commercial spokes for sustainable ruminant innovation worldwide.

This Special Issue showcases the GFP’s capabilities through ten papers from research hub representatives across five continents. Together they illustrate how the hub-and-spoke approach integrates local challenges with global solutions, enabling more sustainable ruminant systems that produce more with less from healthy animals and soils while delivering nutritious, climate-smart food and protecting the environment.

## The Unifying Framework

We open with a paper summarizing the vision and operating model of the GFP itself. In Rivero et al. (2026), the authors articulate how research hubs generate evidence-based practices that are rapidly adapted and disseminated through commercial spokes. From Malawi’s smallholder systems to temperate UK dairy operations, the network rejects one-size-fits-all solutions in favor of bidirectional knowledge flow informed by local practitioners, climate resilience, One Health, and smallholder empowerment. This paper serves as the cornerstone for the entire issue, demonstrating the GFP’s scalability, policy relevance, and alignment with the FAO’s sustainable livestock transformation agenda.

## Foundational Tools for Transformation

The following papers explore the scientific and technological enablers that make the hub-and-spoke approach effective. Adaptive genomics emerges as a powerful toolkit for circular intensification. Elayadeth-Meethal et al. (2026) synthesize GFP-linked research on native cattle breeds (notably the Vechur (*Bos p. indicus*)) and show how adaptive alleles, haplotypes, and the G → P → E → SI (genotype → phenotype → environment → system impact) framework can be harnessed through directed haplotype introgression. The result is low-input, climate-resilient, low-emission herds that maintain productivity, with insights generated at hubs actively ready for rapid deployment at spokes (small holder farmers in Kerala, India).

Long-term agroecosystem research platforms provide the rigorous, real-world evidence base. Cartmill and Busch (2026) describe the Pioneer Farm (PFAR) in Wisconsin, USA, as a hybrid hub-and-spoke infrastructure. Through edge-of-field monitoring, soil-health assessments, farmer-led watershed partnerships, and alignment with Long-term Agroecosystem Research (LTAR) and GFP networks, PFAR demonstrates how polycentric governance and co-production resolve complex soil, nutrient, and water-quality dynamics under working-farm conditions.

Digital innovation and open data are equally transformative. Lewis et al. (2026) detail practical strategies for Findable, Accessible, Interoperable, Reusable (FAIR) data at a GFP hub (Future Farm at Harper Adams University, UK), overcoming barriers of ownership, interoperability, and curation to accelerate innovation across research, education, and commercial partners. This is complemented by the development of digital visions for extensive systems, where new tools enable precise, adaptive management of rangeland grazing in Scotland (Morgan-Davies et al., 2026) and report preliminary lessons from the GLENCOE farmlets on early trajectories of intensified grazing management on basaltic native grasslands in Uruguay (Devincenzi et al., 2026).

## Regional Applications and Holistic Demonstrations

With the framework and tools established, the issue turns to diverse, on-the-ground implementation that address regional challenges while contributing to global knowledge curation. In Africa, forage biodiversity is mobilized as a cornerstone of sustainable livestock systems. The hub-and-spoke model links central scientific capacity with locally driven adaptation, unlocking native legumes and climate-adapted grasses to boost product­ivity, restore soils, regenerate ecosystems, reduce emissions, while supporting equitable rural livelihoods (Akpensuen et al., 2026). In the humid tropics, Silent Valley Farm in Kerala, India, exemplifies a full-scale commercial best-practice dairy system (Murugan et al., 2026). Integrating crossbred and native Vechur cattle, Attappady Black goats, fodder production, silvopastoral systems, automated heat-stress mitigation (“*aswasa*”, meaning relief), carbon farming, water stewardship, biodiversity conservation, and strong community engagement, the farm demonstrates “Clean, Green and Ethical” (CGE) production that simultaneously enhances animal welfare, reduces environmental impacts (emissions and conservation), and strengthens local socio-economic resilience.

In South America, integrated crop–livestock systems are rigorously evaluated for trade-offs. Pereyra-Goday et al. (2026) analyze four rotations at Uruguay’s Palo a Pique long-term experiment and shows that long-rotation systems deliver the best overall balance with highest gross margin, lowest emissions and synthetic nitrogen per dollar of margin, and soil erosion well below tolerance limits; while continuous cropping maximizes short-term food security, but is unsustainable in the long-term. Multi-criteria assessment is shown to be essential for evidence-based policy and producer decisions.

Finally, the Future Farm project at Ridgefield in Western Australia (Martin and Murugan, 2026) provides a compelling multidisciplinary synthesis. Operating as a sheep-crop enterprise in a Mediterranean climate, it tests CGE sheep breeding, novel indigenous shrub forages, conservation cropping, ecosystem restoration, carbon farming, water management, and community initiatives. The farm serves as both a research platform and a beacon for “feeding the world without destroying the planet.”

## Collective Impact and Future Horizons

Across these ten papers, the GFP network is revealed as far more than a knowledge-exchange mechanism. It is a resilient and adaptive ecosystem that:

- generates foundational science (genomics, long-term monitoring, digital systems);
- translates foundational science into context-specific applied innovations; and
- scales those innovations through commercial locally adapted spokes to deliver economic gains, environmental stewardship, and social benefits at scale.

From soil health in the Upper Midwest to forage biodiversity in Africa, heat-stress mitigation in the humid tropics, integrated crop–livestock systems in Uruguay, to holistic sheep-crop systems in Australia. The contributions demonstrate that sustainable ruminant production is achievable when research hubs and practitioner spokes collaborate as equal partners. This Special Issue therefore not only showcases the GFP’s ongoing work, but also offers a practical roadmap for policymakers, researchers, extension services, and producers worldwide. By investing in and expanding such networks, we can accelerate progress toward the Sustainable Development Goals, align with FAO’s vision for sustainable livestock transformation, and ensure that ruminant systems continue to nourish humanity while safeguarding planetary health and delivering on the Paris Climate Agreement. Our hub-and-spoke approach is proven, scalable, and inclusive, and provides the blueprint for that future.

We thank all authors, reviewers, and GFP members for their contributions to this timely and impactful collection.

## Author Biographies

**Dr Jordana Rivero** is a researcher in Grazing Livestock Systems and inaugural Chair of the Global Farm Platform. Her work focuses on livestock grazing systems with an emphasis on sustainability, covering pasture and grazing management, livestock performance, animal behavior and welfare, forage quality, and breeding objectives for several livestock species. She holds an agricultural engineer degree from Uruguay, where she developed breeding objectives for dairy cattle under pasture-based systems, and a PhD in Agricultural Science from the Universidad Austral de Chile, gaining experience in grazing systems and NIRS applied to feeds and animal products. Her postdoctoral research examined the joint effects of agronomic management and perennial ryegrass cultivars on grazing dairy cows’ performance, nutrient use efficiency, and emissions.

**Dr Joshua Onyango** is a Lecturer in Veterinary Science, International Lead and Researcher at Harper and Keele Veterinary School (joint Veterinary school between Harper Adams University and Keele University), United Kingdom, and Deputy Chair of the Global Farm Platform. His research interests broadly focus on animal health and production, One Health, infectious and non-infectious diseases of veterinary and human importance. He studied animal health at Egerton University in Kenya, a BSc (Hons) at University of the West of England, an MSc in Animal Health and Production and a post graduate Certificate in Education both at University of Essex and a PhD in Veterinary Epidemiology from University of Northampton, United Kingdom where he looked at risk factors for uterine infections in English dairy herds and epidemiology of orf in English sheep flocks.

**Professor Tommy Boland** is the Professor of Ruminant Nutrition and Sheep Production at University College Dublin. His research focuses on enhancing the sustainability of pasture-based livestock systems, with a particular emphasis on methane mitigation, multi-species swards, and nutrient use efficiency. He serves as Co-Chair of the Livestock Research Group for the Global Research Alliance and leads the climate-neutral animal agriculture challenge at the BiOrbic SFI Centre. He is also an Adjunct Associate Professor at Massey University. He is a past member of the Livestock Farming Systems Commission of the EAAP and has organized numerous national and international scientific conferences.

**Dr Claire Morgan-Davies** is Reader in Livestock Systems at Scotland’s Rural College (SRUC). Claire is based at SRUC’s Hill and Mountain Research Centre, Kirkton & Auchtertyre research farms, near Crianlarich (Scotland, UK) where she has focused on extensive sheep systems research, knowledge exchange and participatory research, for over 25 years. In the past 10 years, her research has moved on toward technology use, uptake and adoption by small ruminant farmers in the UK and Europe.

**Professor Michael Lee** is Deputy Vice Chancellor at Harper Adams University, England’s premier specialist agriculture and land use university. He is an expert in sustainable livestock systems, with a focus on defining their role in securing global food security while protecting environmental health (Livestock’s role in human and planetary health). He holds a first class honors degree in Animal Science from the University of Wales, Aberystwyth, and a PhD in ruminant nutrition (protein and energy metabolism) from the University of Aberdeen. He also earned a Postgraduate Certificate in Teaching in Higher Education from Aberystwyth University. His research interests center on sustainable livestock production, ruminant nutrition, and the integration of livestock systems with environmental sustainability and food security objectives.
